# More than a Feeling: Dermatological Changes Impacted by Spaceflight

**DOI:** 10.21203/rs.3.rs-2367727/v1

**Published:** 2023-02-10

**Authors:** Henry Cope, Jonas Elsborg, Samuel Demharter, J. Tyson Mcdonald, Chiara Wernecke, Hari Parthasarathy, Hriday Unadkat, Mira Chatrathi, Jennifer Claudio, Sigrid Reinsch, Sara Zwart, Scott Smith, Martina Heer, Masafumi Muratani, Cem Meydan, Eliah Overbey, JangKeun Kim, Jiwoon Park, Jonathan Schisler, Christopher Mason, Nathaniel Szewczyk, Craig Willis, Amr Salam, Afshin Beheshti

**Affiliations:** University of Nottingham; Abzu; University of Oxford; Georgetown University; Technical University of Munich; University of California, Berkeley; Princeton University; University of California, Berkeley; NASA Ames Research Center; NASA Ames Research Center; USRA; NASA Johnson Space Center; Erfurt and University of Bonn; University of Tsukuba; Weill Cornell Medicine; Weill Cornell Medicine; Weill Cornell Medicine; Weill Cornell Medical College; The University of North Carolina at Chapel Hill, Chapel Hill; Weill Cornell Medicine; Ohio University; University of Bradford; King’s College London; Broad Institute of MIT and Harvard

## Abstract

Spaceflight poses a unique set of challenges to humans and the hostile Spaceflight environment can induce a wide range of increased health risks, including dermatological issues. The biology driving the frequency of skin issues in astronauts is currently not well understood. To address this issue, we used a systems biology approach utilizing NASA’s Open Science Data Repository (OSDR) on spaceflown murine transcriptomic datasets focused on the skin, biomedical profiles from fifty NASA astronauts, and confirmation via transcriptomic data from JAXA astronauts, the NASA Twins Study, and the first civilian commercial mission, Inspiration4. Key biological changes related to skin health, DNA damage & repair, and mitochondrial dysregulation were determined to be involved with skin health risks during Spaceflight. Additionally, a machine learning model was utilized to determine key genes driving Spaceflight response in the skin. These results can be used for determining potential countermeasures to mitigate Spaceflight damage to the skin.

## Introduction

Throughout the course of a Spaceflight mission, the astronaut exposome includes altered gravity, elevated radiation, and confinement within a closed environment with limited hygiene procedures and ventilation^1^. These stressors perturb biological systems, inducing gene regulatory changes, mitochondrial dysregulation, microbiome shifts, and DNA damage^1^. Dermatological issues are not typically regarded as a key risk to astronaut health and mission success, yet they are amongst the most common in-flight health issues reported by astronauts. During regular ISS missions averaging 6-months, skin rashes have been identified as the most frequently reported in-flight clinical symptom, with 1.1 cases per flight year, accounting for 40% of all notable medical events and a 25-fold increase compared to the US general population^2^. An additional 0.3 cases per flight year include skin manifestations accompanied by symptoms of infection; skin lesions associated with viral reactivation were also reported and have been studied in-flight^2,3^. Notably, an in-flight skin rash was reported to have occurred during a 6-month ISS mission, worsening immediately after extravehicular activity (EVA)^4^, and a post-flight skin rash was reported following the 1-year long NASA Twins Spaceflight study^5^. These events arouse particular concern for future Moon and Mars missions, which will be longer, include high-levels of EVA, and also include risk of exposure to irritant dust, reported previously to induce skin issues in Apollo astronauts^6^. Elucidating the biological response of skin in space could aid development of new countermeasures, to manage dermatological issues and optimize astronaut performance during these future missions.

As the body’s largest organ, skin serves a myriad of important health functions, including fluid diffusion, wound healing, thermoregulation and tactile sense. Importantly for Spaceflight, skin is also the first line of defense against pathogens and radiation. Studies of the astronaut skin microbiome have identified microbial interchange between the skin and the ISS interior^7^, and have also hypothesized that abnormal proliferation of certain types of opportunistic microorganisms on astronaut skin may stem from the unusual hygiene procedures on the ISS, where wipes are used as opposed to showering^8^. While these microbiome shifts and their associated health effects require further investigation, investigations into the molecular response to skin in space are lacking. In murine skin, the 13-day STS-135 study and the 91-day Mouse Drawer System (MDS) study reported significant Spaceflight-induced modulation of extracellular matrix (ECM) genes^9,10^; the set of genes did not overlap, which could be due to significant differences in study design (e.g. duration). The MDS study also reported a 42% increase in synthesized procollagen, a 15% reduction in dermal thickness, and an increase in hair follicles growing in the anagen stage accompanied by dysregulation of hair follicle genes^10^. Corroborating gene regulatory changes associated with hair cycle were also reported in an analysis of hair follicle samples from 10 astronauts in a JAXA study^11^, yet reports of skin physiological changes in astronauts, including dermal atrophy, have been mixed^12,13^. In a recent analysis of astronaut skin punch biopsies taken before and after the first 3-day commercial Inspiration4 (i4) mission, gene expression changes within different layers of the skin were explored alongside microbiome changes, revealing immunological changes in the inner regions of the skin (i.e., vasculature, outer dermis) and DNA damage and repair changes in the outer epidermal layer of the skin^14^.

In this study, we performed an analysis of five previously unreported murine skin RNA-Seq datasets from the JAXA Mouse Habitat Unit 2 (MHU-2), and NASA Rodent Research 5 (RR-5) and 7 (RR-7) experiments, to identify global signatures in Spaceflight-exposed skin, and to investigate the effect of study design on biological signatures relating to skin health and common Spaceflight themes including DNA damage and repair, and mitochondrial dysregulation^1^. In addition to examining gene regulatory patterns, we used an explainable artificial intelligence (AI) modeling approach to construct interpretable machine learning models to identify synergistic effects between pairs of genes, which we interpret as possible biological interactions that reveal a putative disequilibria of dependent processes. We then compared gene regulatory changes in the rodent data to astronaut data, including JAXA hair follicle data^11^ and data from the NASA Twins^15^, JAXA Cell-Free Epigenome (CFE), and i4 studies. We conclude by suggesting how these molecular signatures may eventually lead to follow-up studies and pharmaceutical interventions.

## Results

### Spaceflight transcriptome global changes in rodent skin reflect common biological hallmarks of Spaceflight

**Figure 1:**
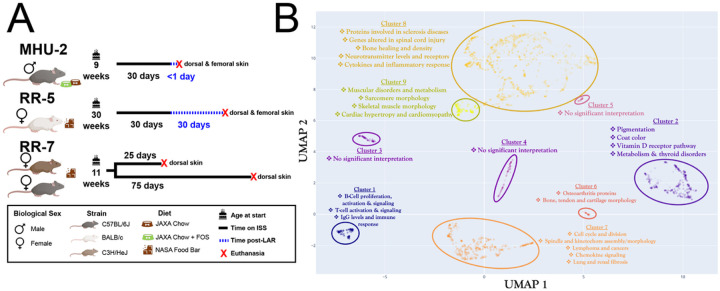
Global data overview. **A)** Breakdown of the rodent datasets used in this study. **B)** Clustering of the most variable genes within the rodent datasets with functional annotation.

To explore whether transcriptomic changes in the skin would occur during Spaceflight we analyzed five RNA-Seq datasets from the NASA Open Science Data Repository (OSDR)^16^, all representing skin tissue from spaceflown mice and matching ground control replicates. These datasets are derived from three different Spaceflight missions (Fig. 1A). The most variable genes across the datasets cluster into functional groups related to established physiological risks of Spaceflight (Fig. 1B). For example, Spaceflight is known to induce immune dysfunction^1^ and Cluster 1 involves highly-correlated genes associated with immune response, including genes linked to modulation of Immunoglobulin G (IgG) levels. Microgravity is also well established to cause degradation of muscle^1^ and Cluster 9 contains a significant number of genes related to muscular morphology and muscle disorders.

**Figure 2:**
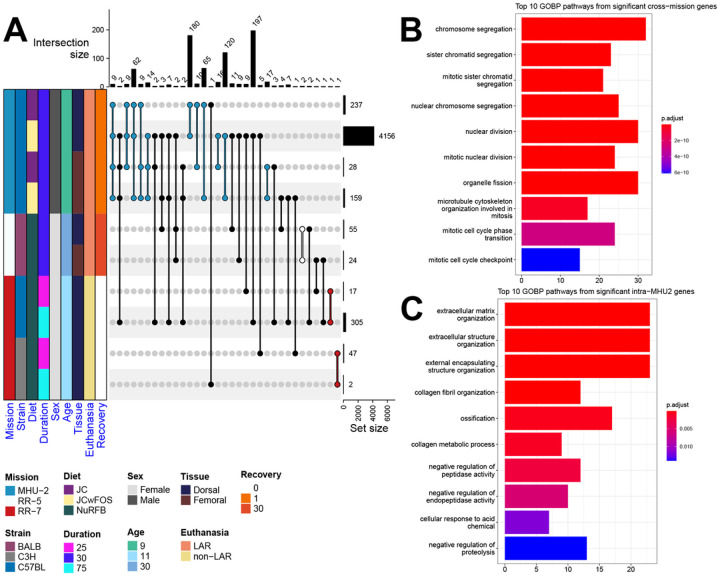
Categorizing overall key pathways and genes being regulated in the skin during Spaceflight. **A)** An upset plot showing the number of significant (FDR ≤ 0.1) DEGs in Spaceflight versus ground data subsets and the number of overlapping significant DEGs between these data subsets. The colored annotation bar on the left of the plot shows how the original datasets divide into 10 data subsets with various conditions including diet, biological sex and strain. The bar plot on the right of the plot shows the number of significant DEGS in each of the 10 data subsets. The bar plot on the top shows the number of intersecting DEGs between combinations of the data subsets, as indicated by the connected dots within the body of the upset plot. Black connecting lines indicate combinations spanning across multiple missions, and other connecting lines are colored according to the annotation bar, based on their mission. **B)** A bar plot of the top 10 most significant GOBP pathways found in the union of cross-mission combinations (i.e., DEGs from the black bars in panel A). **C)** A bar plot of the top 10 most significant GOBP pathways found in the union of intra-MHU-2 combinations (i.e., DEGs from the blue bars in panel A).

Upon subsetting each dataset based on experimental groups (i.e. diet, strain, skin site) and performing differential gene expression (DGE) analysis for Spaceflight samples versus ground control samples, we end up with 10 data subsets (Fig. 2A). In total 476 unique genes are significant (FDR ≤ 0.1) in at least 2 out of the 10 data subsets. One significant gene uniquely shared across data subsets from the RR-7 study is GLYCAM1 which is shared between both data subsets for the C57BL/6J strain. GLYCAM1, which supports lymphocytes in transfer from the bloodstream into lymphoid tissues^17^, is strongly upregulated (LFC ≈ 18.81) at the 25 day time point and strongly downregulated (LFC ≈ −15.66) at the 75 day time point, which could indicate an immune response within the first half of the RR-7 mission for the C57BL/6J mice. Over representation analysis (ORA) reveals that the 247 significant genes uniquely shared between MHU-2 data subsets are associated with the organization of collagen and the ECM (Fig. 2B), with the 225 significant genes uniquely shared across missions primarily associated with cell-cycle processes, including cell division (Fig. 2C). Spaceflight is well-documented to perturb the cell-cycle^1^ and significant modulation of genes associated with ECM homeostasis were previously reported in analyses of the skin of spaceflown mice^9,10^. The MHU-2 subset from the dorsal skin of mice fed a standard JAXA chow diet with supplemental prebiotic fructooligosaccharides (FOS) contains the highest quantity of significant (FDR ≤ 0.1) differentially expressed genes by a substantial margin (Fig. 2A). In ground-based rodent studies, FOS has been shown to improve gut microbiome balance, increase bone density, and affect the immune system through increased short chain fatty acid (SCFA) production (modifying interleukin production and natural killer cell activity), and modification of the immune system via gut-associated lymphoid tissue^18,19^. Thus, we may expect FOS ingestion to increase ECM activity, due to restructuring of the collagenous ECM of bone. Due to this disparity between the number of significant DEGs in the data subsets, and the variety of conditions between data subsets, care must be taken when combining these data subsets to infer shared responses of rodent skin to Spaceflight.

### Key genes involved in rodent skin Spaceflight response are associated with cell cycle regulation and lipogenesis

**Figure 3:**
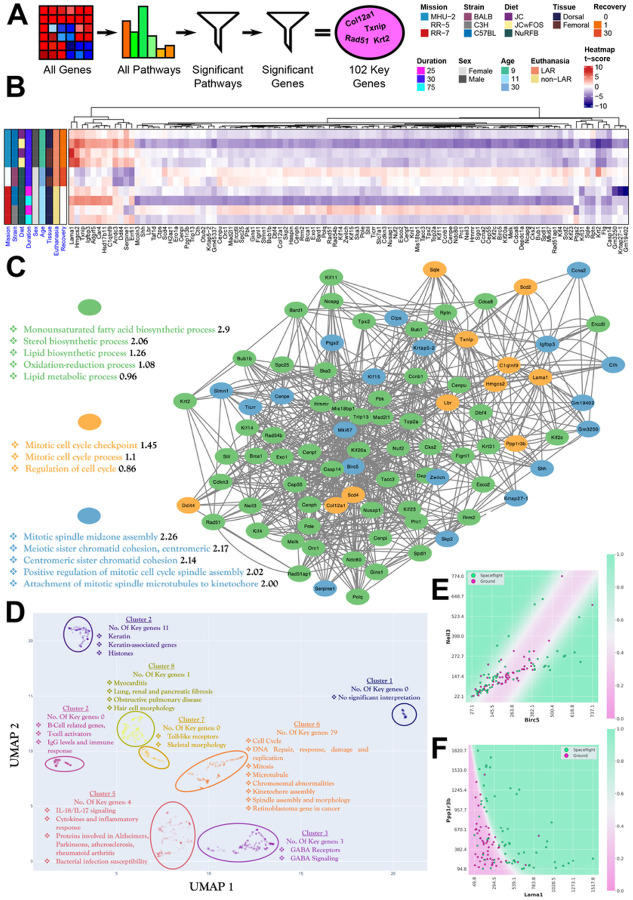
Key genes involved in rodent skin Spaceflight response. **A)** A graphical representation of the method for deriving the set of 102 key genes pathways, where highly significant (FDR ≤ 0.05) pathways in at least 8/10 data subsets were selected and then leading-edge genes that were significant (FDR ≤ 0.1) in at least 2 datasets from different missions were accepted. **B)** A heatmap showing regulatory changes in the key genes within each rodent data subset. **C)** Shows the graph resulting from linking every gene (visualized as a node) to its five top synergistic partners, as described in the main text and the methods section. We also show the three most significant functional clusters obtained through PPI analysis. **D)** Shows a functionally clustered set of 1060 genes, in which all key genes have been manually added to visualize the functional correlations present in the key gene sets and how they relate to other highly variable genes. **E)** Shows the decision boundary of the key gene model with the largest synergistic effect between two genes. **F)** Shows the decision boundary of the model with the highest predictive performance overall using only two genes. Both models have plausible biological interpretations, as outlined in the text.

To characterize the shared response of murine skin to Spaceflight across the datasets, a manageable set of key genes were identified. In an effort to overcome the aforementioned bias of the MHU-2 dorsal skin with FOS-supplemented diet, this set of 102 key genes was derived by performing Gene Set Enrichment Analysis (GSEA) on all genes, and taking the genes involved in significant biological pathways common across the majority (i.e., 8/10) of the datasets, and then further filtering to only include genes that were significant across multiple missions (Fig. 3B). All 102 key genes overlap with the aforementioned 472 significant (FDR ≤ 0.1) genes intersecting between combinations of 2 or more data subsets. For the majority of the genes, genes are significant with a trend of downregulation in the MHU-2 dorsal skin dataset with the FOS-supplemented diet, and in the RR-7 mission C57BL/6J mice after 75 days of flight (Fig. 3B). A smaller collection of genes (*LAMA1*, *HMGCS2*, *TXNIP*, *IGFBP3*, *ADGRF5*, *CAR4*, *HSD17B11*, *C1QTNF9*) are also significant in those same data subsets, but are significantly upregulated (Fig. 3B).

For the most significant genes, convincing correlations exist between Spaceflight status and the up- or down-regulation of a particular gene. For example, in the case of the gene with the lowest p-value, *LAMA1* (Laminin Subunit Alpha 1), the mean gene expression of Spaceflight mice is 2.2 times the mean expression of ground mice, indicating significant up-regulation in mice that went to space. To go beyond single-gene significance, we used QLattice modeling^20^ to derive a quantitative measure of synergy between all possible 2-gene combinations in the set of key genes. For every gene, our method picks out the five best partner genes and we interpret these five partner genes as the ones most likely to have a meaningful interaction with the target gene. In this way, the strengths of the connections become proxies for the interaction strength/likelihood in the actual data, and remains agnostic to interactions known from literature. To visualize the results, we display a network of key genes and their top 5 partners drawn as node connections (Fig. 3C). The more central a gene is, the more synergistic partners the gene has. Subsequently, we can then use external evidence to annotate our findings, which identified three functionally distinct clusters in the network via Protein Protein Interaction (PPI) analysis^21^. In this PPI network, the first cluster primarily contains genes involved with pathways associated with lipogenesis. This family of pathways might be crucial in Spaceflight, as evidenced by previous studies showing the capacity of Spaceflight to alter liver metabolism, resulting in loss of liver function from lipid accumulation and changes to lipid related proteins in murine liver samples^22,23^.

The second cluster primarily consists of genes predictive of cell cycle pathways, with several of the genes (*LAMA1*, *HMGCS2*, *TXNIP*, *C1QTNF9*) clustering with distinct average trends of upregulation across the datasets (Figs. 3C and 3B). These genes tend also to be involved in metabolic pathways, particularly those associated with diabetes; *HMGCS2* has been shown to regulate mitochondrial fatty acid oxidation^24^, while *LAMA1* variations have been shown to be risk factors in type 2 diabetes^25^. Aside from being a protector against oxidative stress, *TXNIP* is similarly implicated in metabolic diseases, and is typically upregulated in diabetic and prediabetic muscle tissue^26^. *C1QTNF9* (also known as CTRP9) has also emerged as a potentially important component of pathways involving lipid metabolism and adipose tissue, exemplified by the fact that *C1QTNF9* transgenic mice have been shown to resist weight gain and metabolic dysfunction^27^. It is interesting to note the significantly lower upregulation of these metabolic pathway genes for female mice. This might be linked to the same mechanisms offering higher protection for females against diet-induced obesity compared to male mice, as has been noted in other studies^28^.

The third cluster (Fig. 3C) relates to cell cycle pathways linked to cell division and microtubules. This also becomes apparent when clustering the key genes with a larger set of 1060 genes (Cluster 6 in Fig. 3D), that contains genes related to cell cycle, cell anatomy/morphology and cell function, including DNA functions. This cluster contains around 80% of the key genes, which indicates that their inclusion in the key genes arise from the fact that cell and DNA functions can be regarded as key functions in Spaceflight. These pathways are often considered to be important in Spaceflight, with studies showing that Spaceflight and altered gravity affects microtubules and mitochondria as well as altering apoptosis in several organisms *in vivo* and *in vitro*^29,30^.

In the synergy network (Fig. 3C), *BIRC5* (Baculoviral IAP Repeat Containing 5) is the gene with the highest node degree, i.e. it is the gene with the most synergistic partners. This does not in itself guarantee that the best performing synergistic gene combination involves *BIRC5*, though in this case it did (Fig. 3E). *BIRC5*, which encodes Survivin, is involved with negative regulation of apoptosis or programmed cell death, when downregulated in cancer cells it has been shown to induce apoptosis and suppress tumor growth^31^. While the *BIRC5*-NEIL3 model attains the highest performance gain when going from one to two model genes, it is not the best-performing model overall. It has an AUC of around 0.73, whereas the best 2-gene model that can be extracted from the key genes has an AUC of 0.85 and an accuracy of 0.82. Thus, the model is correct in predicting mouse Spaceflight status correctly in more than 4 out of 5 cases based on these two genes alone. The model consists of a combination of *LAMA1* and PPP1R3B (Protein phosphatase 1 regulatory subunit 3B) (Fig. 3F).

### Astronaut data correlates with key biological changes occurring in the murine skin models

**Figure 4:**
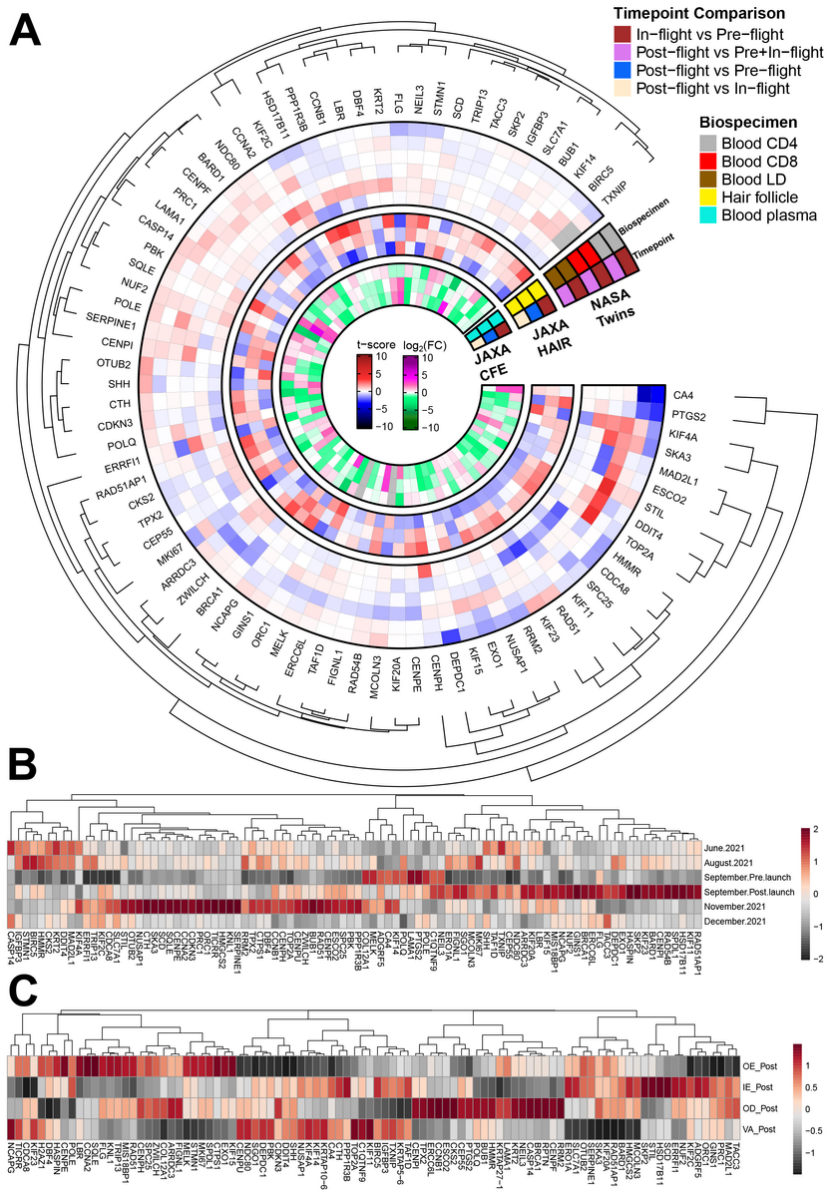
The pro le of the key genes in astronauts. **A)** Heatmap investigating changes in the rodent skin data key genes in astronaut data derived from the NASA Twins, JAXA CFE, JAXA hair follicle data, and Inspiration4 studies. **B)** Heatmap showing average expression scaled in blood PBMCs data from the Inspiration4 mission for different timepoints. **C)** Heatmap showing average expression in skin data from the Inspiration4 mission for different skin layers.

Having established a set of 102 key genes regulated by Spaceflight in rodent skin, we then decided to investigate the expression of these genes in humans. We compared data from the blood of astronauts from the NASA Twins and JAXA CFE studies, astronaut hair follicles from a JAXA mission, and blood and skin from i4 astronauts (Figs. 4A and 4B). In the NASA Twins Study, the most noteworthy results are in CD4 cell type in-flight vs pre-flight samples with significant downregulation in a group of genes correlating positively with murine skin samples, e.g. *CA4* and PTGS2. These genes display no significant changes in CD8 cells, which can indicate that downregulation is a response to immune system stressors affecting the helper function and signaling of CD4 cells. Overall, the effects seen in the NASA Twins Study are mostly constrained to one type of sample, and only two genes have noteworthy changes across all biospecimens (*KIF4A*, *SKA3*).

In hair follicles extracted from JAXA astronauts the most common pattern is genes experiencing in-flight vs pre-flight upregulation, with subsequent post-flight vs in-flight downregulation (e.g. *PPP1R3B*, *CCNB1, HSD17B11, KRT2, GINS1*). In the CFE study, blood samples from JAXA astronauts had the strongest in-flight upregulation in *RAD54B*, *FLG*, *CA4* and in particular in *CASP14*, where upregulation continues post-flight. *FLG*, *RAD54B* and *CASP14* were all genes with general downregulation across the murine data, whereas *CA4* saw general upregulation. This indicates that some genes have similar responses in skin and blood, whereas others see counteractive circulating responses. Another gene of interest to our analysis is *BIRC5*, where in-flight vs pre-flight downregulation persists and neither return to baseline or further downregulation is seen post-flight.

In the i4 mission, blood samples (Fig. 4B) show a consistent pattern of strong post-flight upregulation in several oncogenes, e.g. *BARD1*, *BRCA1*, *RAD54B*, *EXO1*, *SPDL1* and *HSD17B11*. Some of these upregulations were also observed in the murine data. Two months after terrestrial return, several of these genes either returned to baseline or experienced downregulation compared to pre-flight, and a new group of significantly upregulated genes emerged (*SERPINE1, KNL1, HMGCS2, TICRR, ORC1, PRC1, CDKN3, CCNA2, CENPE, SQLE, SCD, SKA3, CTH, NUSAP1, OTUB2*, and *STIL*) related to cell cycle pathways.

We observed an interesting vascular response for the genes from the i4 skin samples (Fig. 4C); genes respond oppositely when comparing the outer epidermis to the vascular samples. Genes *MCOLN3* through *ERO1A* (Fig. 4C) are e.g. strongly downregulated in vascular samples, with mild to moderate upregulation in dermal tissues. This group might have decreased circulating expression as counteracting effects to skin damage, which we elaborate on in the discussion. For skin-specific genes, we saw a moderate upregulation of the gene controlling the production of the protein Filaggrin (*FLG*), which is important for maintaining epidermis structure and consequently plays a role in allergic skin diseases^32^.

### Specific pathways and genes related to skin health are altered in Spaceflight

**Figure 5:**
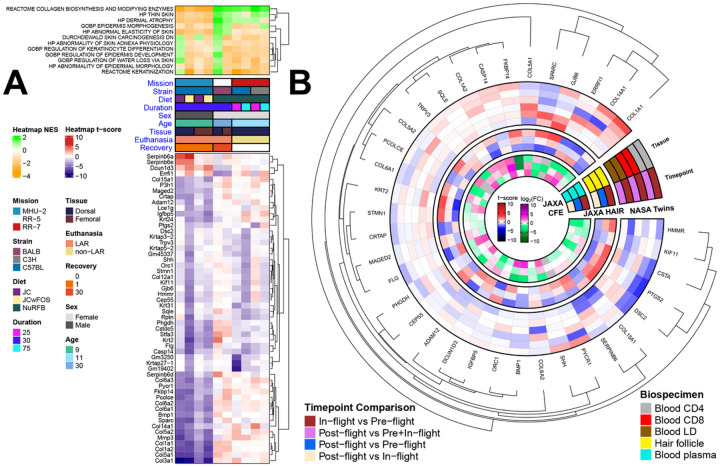
Behavior of the specific genes associated with skin health in astronauts and rodents. **A)** The orange (suppressed) and green (enriched) heatmap shows the normalized enrichment score (NES) of pathways that are significant (FDR ≤ 0.25) in at least 1. The red (upregulated) and blue (downregulated) heatmap shows the t-score for leading edge genes from the significant pathways that are significant (FDR ≤ 0.1) in at least 2 data subsets. **B)** The circular heatmap shows changes from the genes in A in human data.

To determine direct relevance of Spaceflight to skin health we conducted targeted analysis with a curated list of key pathways involved in skin health from MSigDB and identified which of these pathways were significantly modulated in the murine skin datasets. A Collagen biosynthesis pathway and a set of collagen genes (*COL1A1 & COL1A2, COL3A1, COL5A1 & COL5A2, COL6A1 & COL6A2 & COL6A3, COL14A1*) were significantly suppressed/downregulated across all dataset subsets for the MHU-2 mission, while showing a trend of weak enrichment/upregulation across two other missions (Fig. 5A). Overall, the contrasting findings suggest a study design difference in the MHU-2 mission, where the use of young single-housed male mice or dissection shortly after the hypergravity event of LAR may have suppressed collagen biosynthesis. We also find an enrichment of pathways relating to thin skin and dermal atrophy in the RR-5 and RR-7 mice, with suppression occurring in the MHU-2 mice. Several *SERPINB6* genes are significantly downregulated (FDR ≤ 0.1) in the MHU-2 dorsal skin data subset. The RR-5 mission generally lacks any significant results for genes relating to skin health, which could be due to the 30 days of recovery post-flight.

When comparing these results to the astronaut data, similar to *FLG* being downregulated in 9/10 of the murine data subsets, *FLG* is downregulated for in-flight vs pre-flight timepoints for the JAXA hair study, the JAXA Liquid Biopsy study and the CD4 blood cell type from the NASA Twins Study (Fig. 5B). Conversely, *FLG* was upregulated in the outer and inner dermis of the i4 study post-flight vs pre-flight comparison, which could be due to the significantly shorter duration of Spaceflight (Fig. 4C).

### Radiosensitivity of mouse strains impacts DNA damage response following Spaceflight

As part of our targeted analysis, we also opted to investigate the modulation of DNA damage and repair pathways in rodent skin. DNA damage & repair is a well established response to space radiation^15,33^ and little research is done on the consequences this will have on the skin during Spaceflight. When ionizing radiation hits DNA molecules, single-strand breaks (SSBs) and double-strand breaks (DSBs) occur, with DNA Damage Response (DDR) mechanisms activated to repair these breaks^33^. For the majority of DNA damage and repair pathways, both the dorsal and femoral skin RR-5 mission data subsets and the 25 day time point from the C3H/HeJ mice in the RR-7 mission show an opposing pattern compared to the other data subsets. In these three data subsets pathways relating DNA damage and repair are generally enriched, while being suppressed in other data subsets. BALB/c and C3H/HeJ mice, as used in these data subsets, have been shown to be more radiosensitive compared to C57BL/6J mice^34^, so repair mechanisms may be activated to mitigate increased radiation-induced DNA damage. There is a trend of decreasing activity for the DNA DDR pathways from the 25-day to 75-day timepoints which can indicate either adaptation of the DNA repair pathways over time in space or dysregulation following extended-duration Spaceflight. The enrichment of DNA damage and repair pathways in RR-5 follows 30-days of Spaceflight and a post-flight recovery period of 30-days. However, DNA repair genes were reported to still be dysregulated compared to pre-flight levels when evaluated at 6-months post-flight during the NASA Twins study^15^, so persistent DNA damage and repair activation during post-flight recovery is expected.

### Mitochondrial dysregulation increased in the skin during Spaceflight

**Figure 7:**
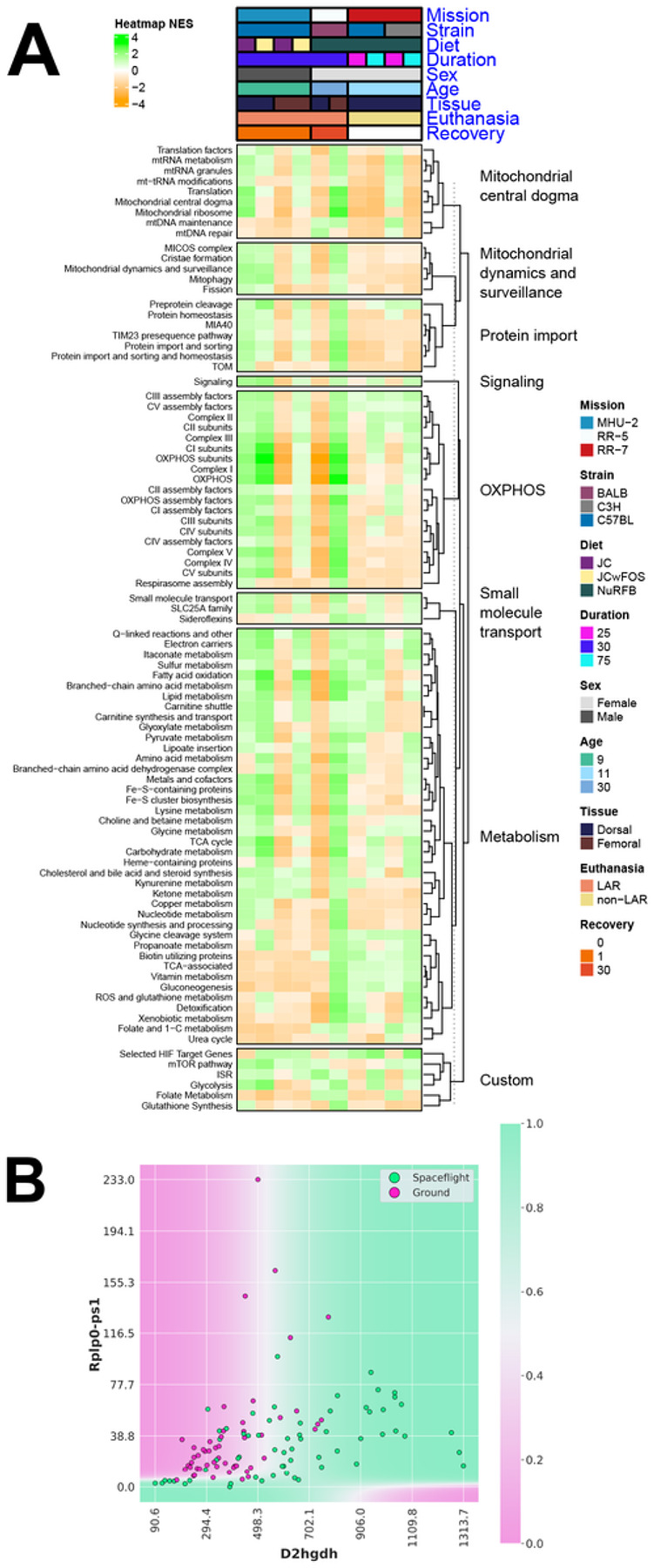
Mitochondrial specific analysis on rodent Spaceflight skin tissue. **(A)** Heatmap of pathways relating to the mitochondria, highly significant (FDR ≤ 0.05) in at least 1 data subset. **(B)** Decision boundary for the 2-gene model related to mitochondrial changes. The model indicates an increased removal of the toxic D2H compound in mitochondria through upregulation of *D2HGDH*, which is less pronounced when PPP1R3Bexpression is suppressed.

Mitochondrial stress has been identified as a key hub for Spaceflight response in multi-tissue analysis, yet skin was not included^30^. Skin is a tissue with high turnover and energy requirements^35^, so we decided to investigate Spaceflight changes relating to mitochondrial stress in murine skin. We found that Spaceflight significantly alters mitochondrial pathways in the skin (Fig. 7), as previously observed in other tissues and across species^30^. Enrichment of an integrated stress response (ISR) pathway in the majority of the datasets (Fig. 7A) is consistent with a previous report of potentially activated ISR due to mitochondrial dysfunction in spaceflown mice^30^. Interestingly, mice that were sacrificed on the ISS without any recovery period on Earth, had an overall suppression of the majority of the OXPHOS complexes which will also be associated with the increased ISR pathways^36^. Additionally, we ran the QLattice machine learning model on the full gene set to obtain a set of models that were unbiased by feature selection due to e.g. variance filtering and the selection of key genes. One of these models demonstrated how the upregulation of *D2HGDH* (D-2-hydroxyglutarate dehydrogenase) synergizes with the downregulation of *RPLP0-PS1* (a gene coding for the ribosomal protein RPLP0) (Fig. 7B)^37^. The upregulation of *D2HGDH* generally indicates an increased ability to break down the toxic D-2-hydroxyglutarate (D2H) compound in the mitochondria. In our case, this could be a compensatory mechanism due to larger build-up of the compound, and the model indicates that this is further dependent on the abundance of the RPLP0 protein.

### Circulating physiological markers from astronauts indicate that exercise countermeasures may have improved skin health

**Figure 8:**
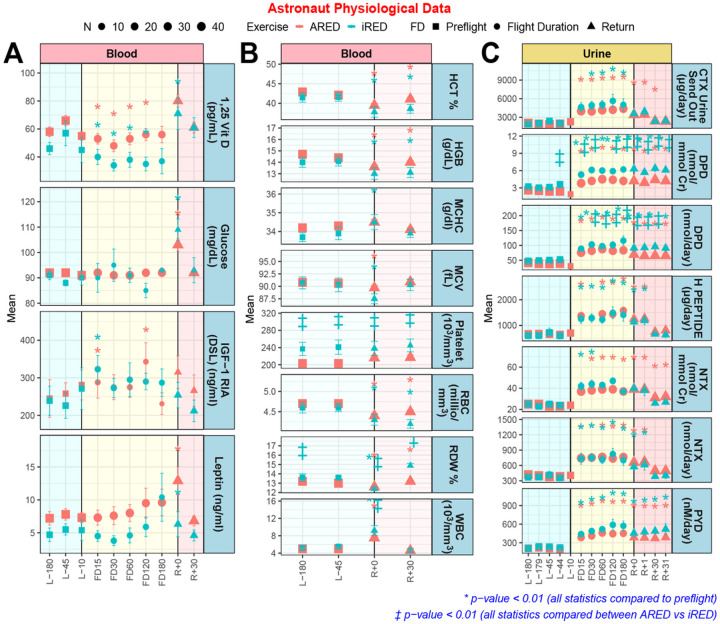
Astronaut physiological markers compiled from up to 50 astronauts. **A)** Specific blood markers which contain data points for pre-launch (L−), flight (FD), and return to Earth (R+). The numbers on the x-axis axis indicate the number of days for each group. Interim Resistive Exercise Device (iRED) is shown in blue and Advanced Resistive Exercise Device (ARED) is shown in red. **B)** Specific blood markers which contain data points for pre-launch (L−) and return to Earth (R+). **C)** Specific urine markers which contain data points for pre-launch (L−), flight (FD), and return to Earth (R+). The statistics on the data are * p < 0.001 for significantly different from L-45 and ‡ p < 0.01 significantly different from ARED.

We investigated standard physiological biomarkers collected from astronaut urine and blood^38,39^ to see whether average trends in these biomarkers connect to Spaceflight gene regulatory changes occurring in the skin (Fig. 8). In-flight increases in IGF-1, leptin and white blood cell levels may indicate altered stress response due to Spaceflight. Following a hypothesis that improved countermeasures may improve skin physiological parameters in astronauts on the ISS^12,13^, we split the data into astronauts that used the older Interim Resistive Exercise Device (iRED), and astronauts that used the newer Advanced Resistive Exercise Device (ARED). Notably overlapping data to that shown in Fig. 8 have been used as evidence of improvements in parameters relating to bone mineral density^38^. Indeed, while vitamin D decreased in-flight and normalized upon return to Earth for both exercise devices, ARED appeared to reduce this drop.

### Vitamin D and L-asparaginase emerged as potential countermeasures for targeting Spaceflight skin dysfunction

**Figure 9:**
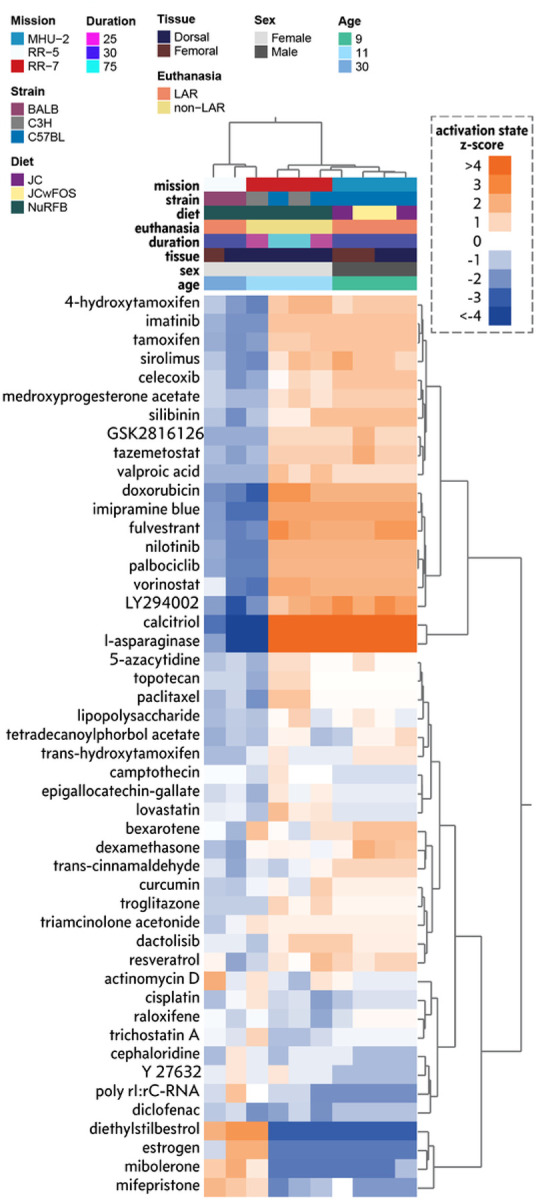
Predicted potential countermeasures for mitigating spaceflight response to the skin. Predicted drug signatures using the key genes across each dataset represented by a hierarchically clustered heat map. A positive (orange) activation state implies key gene expression changes are consistent with mRNA expression changes observed with the indicated drug from curated causal gene expression relationship studies. A negative (blue) activation state implies the key gene expression changes are opposite to mRNA changes observed with the indicated drug.

Having established a key list of genes that changed in the rodent skin, we investigated potential drug targets for these genes via Ingenuity Pathway Analysis (IPA) (Fig. 9). While no consistent potential drug candidates were identified for all of the data subsets, Calcitriol and L-asparaginase exhibited significant activation scores for the majority of the datasets, including all of the data subsets with minimal post-flight recovery period. Calcitriol is the active form of Vitamin D, of which oral supplementation is typically used to prevent low calcium levels and bone disease and topical usage is used to treat plaque psoriasis by inhibiting skin cell buildup, and by decreasing the activity of immune cells in the skin^40^. While Vitamin D supplementation is already used on the ISS^38^, L-asparaginase is a more unexpected target for Spaceflight dysfunction. It is injected to treat acute lymphoblastic leukemia and lymphoblastic lymphoma by depriving leukemic cells of circulating asparagine, leading to cell death^41^.

## Discussion

Skin is well-established as an essential organ for health on Earth, yet despite the frequency of dermatological issues in astronauts^2^, the molecular response of skin to Spaceflight is understudied (see Table S1 for the up-to-date Spaceflight associated skin literature). Here, we have performed a comprehensive study on the impact of Spaceflight on skin, with the aim of addressing gaps in the understanding of Spaceflight associated skin health risks. Our analysis indicated intriguing biological changes occurring in the skin from different mouse strains that provide similar changes occurring in astronauts.

The downregulation of collagen genes in MHU-2 (Figs. 3 and 5) mirrors previous studies wherein a decrease in collagen synthesis was found as a result of hypergravity in cultured human broblasts^42^. In contrast, RR-5 (30-day recovery) and RR-7 (frozen in space) mice had upregulated collagen expression, which is in line with analysis from the same cultured human fibroblast study reporting a 143% increase in collagen synthesis during microgravity^42^. The recovery period of RR-5 mice may reflect terrestrial recovery of collagen expression, but we note that the BALB/c strain is radiosensitive, while the C56BL/6 strain is radioresistant^34^, so although the BALB/c mice in RR-5 received a low duration-dependent dose of radiation, it is likely that radiation-induced changes will be more severe. Increased collagen gene expression may also represent compensatory increases in the production of procollagen, as noted in analysis of the Spaceflight MDS study where an increase in 2 matricellular proteins known to stimulate collagen synthesis in mice skin were found; CTGF and CCN2^10^. The dermal atrophy observed in the MDS study was hypothesized to be connected to early degradation of newly formed, perhaps defective, procollagen molecules^10^.

The difference between missions may also be confounded by sex; all MHU-2 mice are male, while RR-5 and RR-7 mice are female. Mouse studies have shown that the male dermis is thicker than the female, whereas the epidermis (top layer) and hypodermis (subcutaneous fatty layer) are thicker in the female, resulting in skin that is altogether 40% thicker in the male^43^. Skin also generally reacts to circumstantial changes, and studies have found that dermal broblasts *in vitro* sense and react to changes in their mechanical environment^44^ by altering their metabolic activity^45^. Skin samples from male spaceflown mice have previously shown thinning of the dermis due to reductions in dermal thickness and collagen^10^.

There were no significant skin marker changes in RR5 (Fig. 5), but it should be noted that overall no statistically significant skin health markers changed in these missions, which might be due to the 30 days of post-flight recovery. The genes that do not return to basal levels are naturally those of gravest concern in this case, of which *DCUN1D3*, a cell cycle/survival gene typically expressed in tumor tissues, was the only one. This terrestrial return to baseline indicates that in-flight changes are acute responses to the changing environment, which is in line with the acute stress response and cortisol surge previously observed in rodents exposed to hypergravity events^46^ and astronauts exposed to Spaceflight^47^. Such responses are also seen elsewhere in skin; dermal fibroblasts that are exposed to cotricotropin-releasing (CRH) hormone increase proopiomelanocortin (POMC) gene expression and ACTH production which stimulate the production of corticosterone^48^. This fits well with our observations, since the hypothesized hypergravity-induced surge in cortisol in MHU-2 may then be linked to the downregulation of collagen genes.

In the human physiological data (Fig. 8), we also noted changes in stress-responsive markers, e.g. leptin, which is normally downregulated in response to stress^49^, which contrasts the leptin upregulation seen upon re-entry to Earth. Similarly, white blood cell levels are higher on re-entry, which may be due to leukocytosis - a response that can occur as a result of physical and emotional stress^50^, as seen in overexertion, seizures, anxiety, anesthesia and epinephrine administration. IGF-1 increases are another well-known stress response, but we noticed higher levels during flight than pre-flight. IGF-1 is, however, known to increase with exercise. Accordingly, the results may indicate that the astronaut exercise regimen is effective. Additionally, we noted the higher levels of Vitamin D in astronauts using the ARED device (Fig. 8A). Exercise has been shown to mobilize Vitamin D from fat stores^51^ via upregulation of VDR expression in muscles and to increase circulating vitamin D. Other studies have shown that exercise increases the serum’s 25(OH)D_3_ concentration in young trained boys, and our results support the hypothesis that muscles may both store and release 25(OH)D_3_^52^. This mechanism is likely activated by the stimulating exercise afforded by the > 600lbs loading in the ARED device, and may also be the reason for overall better urinary markers observed in astronauts using this device (Fig. 8C).

The higher glucose levels seen upon re-entry to Earth (Fig. 8C) may be linked to the hyperglycemic state induced by the body under stress in an effort to mobilize energy stores^53^. Conversely, in-flight blood glucose levels were lower, which might be related to the best performing 2-gene model for the key genes *LAMA1* and PPP1R3B (Fig. 3F). *LAMA1* is important in the formation of laminins which are involved in metabolic tissue signaling^54^, and a 2018 study showed that high blood glucose concentrations down-regulates the expression of *LAMA1*^55^. The PPP1R3B gene is the regulatory subunit for the activity of protein phosphatase 1, which activates glycogen synthase and limits glycogenolysis^56^. The expression level of *PPP1R3B* thus correlates blood glucose levels, which fits well with the observation that *LAMA1* upregulation is less significant for spaceflown mice with high *PPP1R3B* expression. Furthermore, it has been reported that transgenic overexpression of *LAMA1* can mitigate muscle wasting and paralysis in mouse models of congenital muscular dystrophy type 1A^57^, which is a plausible counter-active role for *LAMA1* in muscular dystrophy due to microgravity.

This is in line with an observed pattern of counteractive mechanisms observed in Spaceflight, where the upregulation of *D2HGDH* (Fig. 7B) indicates a larger need for breakdown of toxic D-2-hydroxyglutarate (D2H) molecules in mitochondria. In this model, the partner gene RPLP0-PS1 codes for the ribosomal protein RPLP0 and is down-regulated in space mice. RPLP0 has previously been implicated in the top significant network for the mitochondria-mediated cycle of Alzheimer’s disease^58^. Low or absent levels of RPLP0 seem to limit the compensatory mechanism in the model (Fig. 7B). If this occurs, build-up of D2H and suppression of certain enzyme functions can result, causing DNA and histones to enter hypermethylated states and activate oncogenes and suppress tumor suppressors^59^. Since many key genes are linked to effects on tumor progression and metastasis, the build-up of D2H in the mitochondria may provide an explanation for cell changes demonstrated by the model involving *BIRC5* and NEIL3 (Fig. 3E). We note that NEIL3 is important for cell proliferation, with increased expression seen in highly replicative tissues such as bone marrow and cancerous tissues^60^. *BIRC5* is a well-studied apoptosis inhibitor^61,62^, and from these functions our data indicated (Fig. 3E) a perturbed apoptosis-proliferation balance for Spaceflight mice, which we speculate to be due to cell and DNA damage from space radiation.

Overall, the themes of DNA repair and cellular health in the Twin Study corroborate the centrality of cell cycle genes in the murine skin samples, where *BIRC5* was also the single best synergistic partner for a significant number of genes (i.e. *CENPH, KIF14, CDKN3, PRC1, NUSAP1* and *RAD51*, and *NEIL3*) (cluster 6 in Fig. 3D). These genes are also in the identified lipogenesis cluster (Fig. 3C), and the centrality of *BIRC5* may be connected to its role in maintaining adipocytes in response to inflammatory stress^63^, which is likely connected to the protective effect of *BIRC5* upregulation in human adipocyte-derived stem cells in obese patients^64^. *BIRC5* is also downregulated in the flight JAXA liquid biopsy data, whereas *CASP14*, another important apoptosis inhibitor, is strongly upregulated (Fig. 4A).

*CASP14* was one of only few genes with downregulation across all murine skin subsets (Fig. 4b), as well as in the JAXA hair follicles. This is interesting due to the role played by *CASP14* in formation of the skin barrier as well as in skin diseases such as ichthyosis, psoriasis and melanoma^65^. The strong upregulation seen in JAXA liquid biopsies could be hypothesized to counter downregulation in hair follicles and skin by increasing circulation of *CASP14*. This might also be the case for *FLG*, where downregulation was seen in-flight for both the liquid biopsies and hair follicles (Fig. 4A). A subtle difference exists between the two specimen types, however, since the liquid biopsies have *FLG* upregulation in both post-flight vs pre-flight and vs in-flight, whereas upregulation in the hair follicles was only seen in post-flight vs in-flight. The link between filaggrin deficiency and atopic dermatitis is well-known^32^. Since *FLG* downregulation was also seen in the skin samples from Spaceflight mice, we hypothesize that the increased circulation of *FLG* may be a response to repair skin cell damage, where filaggrin production is impaired. The i4 data also contained a group with strongly downregulated genes in the vascular samples where a similar mechanism could be at play for e.g. *SERPINE1* (Fig. 4B and 4C). Downregulation of this gene has been shown to accelerate skin wound healing^66^ while overexpression of *SKA3* and *KIF20A* (which was also observed in this group) has been shown to predict poor outcomes in melanoma patients^67,68^.

In conclusion, we have provided a comprehensive study on the impact of Spaceflight on skin health. Currently there is a gap in knowledge for how space radiation and microgravity affect skin biology, yet many of the common themes of Spaceflight dysfunction emerged in our analysis, suggesting that skin could be an easily-accessible candidate for studying the biological impact of Spaceflight. Our unbiased systems biology analysis revealed some genes of interest and potential countermeasures that can be targeted and utilized in future studies. We believe with our study we have started to address the current gaps and provided some clues on how to potentially mitigate the adverse effects of the space environment to the skin.

### Limitations Of Study

The rodent datasets used come from three different Spaceflight experiments with a variety of confounding variables associated with differences in study design. This means that direct comparison between the experiments is challenging, but indeed this also presents an opportunity to hypothesize how these factors may influence biology, as done in this manuscript. A lack of physiological data, such as dermal thickness, from the rodent datasets means that generated hypotheses relating to mouse physiology cannot be confirmed without follow up investigations, but the use of astronaut data helps translate the results to human relevance.

## Methods

### GeneLab Spaceflight murine datasets

Five RNA-Seq datasets (OSD-238, OSD-239, OSD-240, OSD-241, OSD-254) were downloaded from the NASA OSDR (https://osdr.nasa.gov/bio/repo) via the API, and full dataset descriptions can be found on the dataset pages. These datasets are derived from murine skin from the MHU-2, RR-5, and RR-7 Spaceflight experiments. For MHU-2, singly-housed male C57BL/6J mice were 9 weeks of age when flown on the ISS for 30 days; they were euthanized less than 1 day after return to Earth. Dorsal and femoral skin samples were extracted from MHU-2 mice, and 6 replicates in Spaceflight microgravity and the matching 6 replicates in the 1G ground control were split into 2 sets of 3, with half fed a JAXA chow diet and the other half fed JAXA chow with supplemental FOS^18^. For RR-5, 30-week-old BALB/c mice in shared housing were flown on the ISS for 30 days; following return to Earth, mice were given 30 days to recover before euthanasia and extraction of dorsal and femoral skin tissue. Finally, for RR-7, 11-week-old C57BL/6J mice and C3H/HeJ mice were flown on the ISS for either 25 or 75 days before being euthanized on-orbit.

### RNA-Seq analysis of rodent datasets

Raw counts data, derived via a previously reported pipeline^69^, were downloaded from the NASA OSDR. ERCC genes were filtered out, as were genes with low counts across all samples (<10). DGE analysis using the Wald significance test, and was performed on Spaceflight microgravity and ground control samples using the R package DESeq2^70^ (v1.32.0). For each data subset, microgravity Spaceflight samples were contrasted with ground control samples and cook’s cut off and independent filtering were not used. The R Package biomaRt (v2.48.3) was used to map ENSEMBL gene ids to gene symbols and HGNC symbols. All heatmaps were generated using the R package ComplexHeatmap (v2.9.4) and circular heatmaps were created using a slightly modified version of the R package Circlize (v0.4.15), to support cosmetic changes.

### Pathway analysis of rodent datasets

For ORA, the R package clusterProfiler (v4.0.5) was used on vectors of genes and bar plots were made via ggplot2 (v3.4.0). For GSEA, the R package FGSEA (v1.18.0) was used. GSEA was performed on all DEGs for all data subsets, with rank vectors consisting of HGNC symbols and corresponding t-scores in the data subset. For GSEA performed prior to derivation of the key genes, the skin health pathways and genes, and the DNA damage repair pathways, MSigDB human collections H, C2, and C5 were used (v7.4) and set sizes >15 were permitted. For the mitochondrial pathway analysis, human pathways from MitoPathways (v3.0) were used and set sizes >1 were permitted. To filter to pathways during derivations of the key genes, pathways that were highly significant (FDR ≤ 0.05) in at least 8/10 data subsets were selected (8/10 was chosen based on the experimental supplemented diet in the MHU-2 mission), and then leading-edge genes that were significant (FDR ≤ 0.1) in at least 2 datasets from different missions were accepted. All heatmaps were generated using the R package ComplexHeatmap (v2.9.4)

### RNA-Seq data analysis on Twin Study samples

Longitudinal samples were collected from a male astronaut aboard the ISS and his identical twin on Earth during a 340 day mission including 6 months preflight and 6 months postflight follow-up, for a total of 19 timepoints for the flight subject and 13 timepoints for the ground subject. Blood was collected using CPT vacutainers (BD Biosciences Cat # 362760) per manufacturer’s recommendations. For full details of sample separation and processing see^15^. Briefly, samples collected on ISS were either frozen in −80°C after separation of mononuclear cells by centrifugation (referred to as CPT), or returned to Earth in 4°C in a Soyuz capsule and sorted into CD4, CD8, CD19 populations and a lymphocyte depleted (LD) fraction. Samples collected on Earth were either frozen for mononuclear cells or processed when fresh for sorted cell populations. To correct for the effects of ambient temperature exposure on RNA (approximately 36 hours including landing and repatriation) control samples were created by simulating similar conditions to those that may occur during the ambient return and were compared to fresh blood collections from the same individual. RNA extraction, library prep and sequencing were completed per^15^ using both ribodepletion or polyA selection kits.

Generated sequences were trimmed using Trim Galore! (v0.4.1) and quantified to genes using kallisto^71^ on ENSEMBL transcripts. Differentially expressed genes were called using DESeq2^70^ on each cell type separately by comparing preflight, in flight and postflight groups, controlling for the normal biological variance within the 24 months using the longitudinal data of the ground twin and using the simulated ambient control samples as another covariate for sorted cells^70^.

### JAXA Cell-Free Epigenome (CFE) Study RNA quantification data

Aggregated RNA differential expression data and study protocols were shared through the NASA OSDR with accession number: OSD-530^72^. Plasma cell-free RNA samples for RNA-seq analysis were derived from blood samples collected from 6 astronauts before, during, and after the Spaceflight on the ISS. Mean expression values were obtained from normalized read counts of 6 astronauts for each time point. Heatmaps were made for the 21 genes key genes on the normalized values per time point using R package pheatmap (v1.0.12).

### JAXA astronaut hair follicle data

Gene expression data from 10 JAXA astronauts’ hair follicles^11^ was downloaded from the NASA OSDR (OSD-174). Raw data for 60 total samples was processed using LIMMA with R/bioconductor^73^. Briefly, duplicate sample single-color Agilent microarray data was background corrected, filtered for low expression probes, and quantile normalized. Differential gene expression was measured between pre-flight, in-flight, and post-flight data points using p-values adjusted for False Discovery Rates (FDR) with the Benjamini–Hochberg method.

### Inspiration4 (i4) astronaut sample collection

Four civilians, two males and two females, spent three days in Low-Earth Orbit (LEO) at 585 km above Earth. The mission launched from NASA Kennedy Space Center on September 15th, 2021 and splashed down in the Atlantic Ocean near Cape Canaveral on September 18th, 2021. Several human health and performance related experiments were carried out in collaboration with SpaceX, the Translational Research Institute for Space Health (TRISH) at Baylor College of Medicine (BCM), and Weill Cornell Medicine. Experiments were performed in accordance with the relevant guidelines at the principal investigators’ institutions. Moreover, the different study designs and the corresponding methods to collect and analyze the biological samples were approved by BCM IRB. All biological data derived from the Inspiration4 mission were collected pre and post flights. For this study, only data from blood samples and skin biopsies were used. Pre-flight samples were collected at L-92, L-44, and L-3 days prior to launch to space. Upon return, post-flight samples were collected at R+1, R+45, and R+82 days.

Blood samples were collected before (Pre-launch: L-92, L-44, and L-3) and after (Return; R+1, R+45, and R+82) the Spaceflight. Chromium Next GEM Single Cell 5’ v2, 10x Genomics was used to generate single cell data from isolated PBMCs. Subpopulations were annotated based on Azimuth human PBMC reference^74^.

For skin spatial transcriptomics data, 4mm diameter skin biopsies were obtained from all Inspiration4 crew members, once before flight and as soon as possible after return (L-44 and R+1). These biopsies were ash frozen and processed with the NanoString GeoMx platform. Based on staining images using fluorescent antibodies, a total of 95 freeform regions of interest (ROIs) were profiled across outer epidermal (OE), inner epidermal (IE), outer dermal (OD) and vascular (VA) regions. GeoMx WTA sequencing reads from NovaSeq6000 were compiled into FASTQ files corresponding to each ROI and converted to digital count conversion files using the NanoString GeoMx NGS DnD Pipeline. From the Q3 normalized count matrix that accounts for factors such as capture area, cellularity, and read quality, the DESeq2 method was used to perform DGE analysis.

### Astronaut Physiological data

Data are reported from three human subject experiments conducted on the International Space Station: Nutritional Status Assessment (2006–2012), Dietary Intake Can Predict and Protect Against Changes in Bone Metabolism During Space Flight and Recovery (Pro K) (2010–2015), and Biochemical Pro le (20132018). All protocols were reviewed and approved by the NASA Institutional Review Board and all subjects provided written informed consent.

These missions were 4–6 months in length, and these studies included blood and urine collections before, during, and after flight, with analysis of an array of nutritional and biochemical markers. Blood and urine samples were collected 2 or 3 times before flight: approximately Launch minus (L−) 180 days and L-45 days. In some cases, a third blood sample was collected (typically along with the L-45 collection), and these tubes were centrifuged and frozen for aliquoting after flight batched with the samples collected inflight. Blood samples were collected inflight, at approximately Flight Day (FD) 15, 30, 60, 120, and 180. Postflight samples were collected in the first 24-h after landing (designated return+0, R+0) and again 30-d later (R+30). The R+0 samples were not necessarily fasting, given the time of day and nature of return from flight. Of the 59 crewmembers reported herein: 8 returned on the Space Shuttle, with blood collection 2–4 hours after landing; 51 landed in Kazakhstan, with 7 of them returning to Star City, Russia, with blood collection 8–10 hours after landing; 44 were transported directly back to the Johnson Space Center in Houston, with blood collection approximately 24-h after landing. Pre and postflight collections included two 24-h urine collections, and inflight collections included one 24-h urine collection. These collection techniques have been previously described^75^.

We report here vitamins and metabolites, oxidative stress and damage markers, inflammatory markers and cytokines, liver enzymes and endocrine indices. These were analyzed using standard techniques as previously reported^76^.

As of this writing, data were available for 59 crewmembers (47 male, 12 female). Age at launch was 47.0 ± 5.6 y, body mass at launch was 79.2 ± 11.8 kg (M: 83.3 ± 9.3; F: 63.0 ± 4.5). Body mass index was 25.5 ± 2.9 kg/m2 (M: 26.4 ± 2.6; F: 22.3 ± 1.5).

All available data are reported here, although the reported n for any given test or session varies for a number of reasons, including: not all experiments had all analytes included, mission length differences for some crewmembers, schedule or other issues occasionally precluded sample collection, and methods changes over time. Repeated measures analysis of variance was conducted to test for differences during and after flight compared to preflight, and comparisons among time points were made using a Bonferroni t-test. Multiple comparisons were accounted for, and only those tests with p<0.001 are reported. The data was plotted using R package ggplot2 (v3.3.5).

### QLattice symbolic regression modeling

We used symbolic regression (QLattice v3.0.1^20^) to construct both single-gene models and models involving combinations of synergistic genes which map from the gene expressions to Spaceflight status. For these models, we only distinguish between mice that went to space and mice that didn’t. Conventional statistical methods typically allow for calculating the effect of a single gene at a time through metrics such as p-values and false discovery rates. In contrast, symbolic regression models can reveal combinations of genes and modules that best predict Spaceflight status. These could be linear combinations involving two or more genes that have previously also been shown to be statistically significant, or it could be non-linear combinations that reveal features that on their own were nonsignificant but in synergy with a second feature become highly predictive. In addition, known biological functions of genes included in models, as well as the resultant mathematical relationship between them, can potentially be interpreted to reveal regulations or interactions that are affected by Spaceflight.

In biological pathway analysis, it is well-known that up- or down-regulation of one gene can have cascading effects such that the function of one gene becomes sensitive to that of another^77^. It has previously been demonstrated that parsimonious machine learning models are able to provide accurate outcome prediction in omics data, while preserving interpretability^78,79^. The interpretability often results from the fact that models might demonstrate otherwise opaque relations which become clear when combined effects are taken into account. An example could be an interaction where the regulation of a single gene is itself unimportant for functional changes, *unless* another gene is simultaneously regulated. This would indicate a synergistic compound effect, which naturally can expand to a 3-, 4- or 5-gene synergy and beyond. The combined effect of two or more individually insignificant gene expression levels may thus theoretically be more powerful than the effect of a single gene with low p-value. Thus, traditional statistical analysis masks such effects by the use of metrics related to the individual gene.

### UMAP dimensional reduction and gene clustering

For the clustering of genes shown in Fig. 2A, we performed a uniqueness filtering by first dropping all genes where more than 1/4 of samples had identical expression levels. Subsequently, we filtered out all genes with a variance of less than 1.7, resulting in 2184 filtered genes with high variability. Similarly, prior to the clustering in Fig. 2D, we filtered out all genes with a variance of less than 2.5, to which we manually added all key genes that were not present in this set (all but 4), resulting in a final set of 1060 genes for clustering analysis. The rationale behind the stricter variance filtering in Fig. 2D was that it increases the relative frequency of key genes in the clustering, and allows for a higher resolution in the final plot. Thus, the 102 key genes comprise roughly a tenth of the genes included in Fig. 2D, allowing for a detailed analysis of the ontological themes present in the key gene set.

In both clustering figures, a dimensional reduction was performed subsequent to filtering by uniformly distributing the filtered data on a Riemannian manifold, using the Uniform Manifold Approximation and Projection for Dimension Reduction (UMAP). UMAP is a general purpose manifold learning and dimension reduction algorithm which is similar to t-SNE in that it predominantly preserves local structure. Yet, UMAP preserves more global structure, which makes it a more suited algorithm when the objective of the dimensional reduction is more than simple visualization (in this case the objective is clustering). Genes were then clustered using the Hierarchical Density-Based Spatial Clustering of Applications with Noise (HDBSCAN)^80^. A primary advantage of HDBSCAN is that it is always deterministic for the same hyperparameters, and will thus always return the same clustering, all else being equal. In addition, comparable clustering algorithms such as k-means do not perform well unless clusters are of equal size and density with few outliers. With biological data such as gene expressions, we expect large variation in cluster size and density, making HDBSCAN the ideal choice.

Once genes were clustered, the gene sets belonging to each cluster were extracted and analyzed using three ontology databases in the Python implementation, GSEAPY, of the Gene Set Enrichment Analysis^81^ tool Enrichr^82^. We used the Elsevier Pathway Collection, the 2021 WikiPathway Collection, and the 2021 MGI Mammalian Phenotype Level 4 Collection. These enrichment analyses were used to provide context to each cluster by appending an annotation if a notable amount of hits showed up for a particular association. Cluster 2 in Fig. 2B, for example, consists of 59 genes, of which 10 are found as hits for associations to decreased IgG1 level in the MGI Mammalian Phenotype Level 4 database from 2021, as well as more than 20 hits for B-cell receptor signaling in the Elsevier Pathway Collection, and thus these annotations are appended in Fig. 2B. In contrast, Cluster 0, consisting of 47 genes, has no more than one hit for any association in any database, and is thus deemed to have no significant interpretation.

### QLattice synergy network

For the models involved in constructing the QLattice (https://pypi.org/project/feyn/) synergy network in Fig. 2C, we generally used the “Area Under Curve” (AUC) measure as our performance gain indicator. The AUC can in this case be interpreted as the probability that a classifier will be able to correctly distinguish a Spaceflight mouse from a ground mouse for two randomly chosen data samples; one of each type.

To make the synergy network, we first made a list of AUCs for 102 single-gene models for spaceflight using the QLattice; one for each key gene. Subsequently, we constructed 101 2-gene models for each of the 102 key genes by combining it with each of the 101 remaining genes in a QLattice model. We emphasize that for each gene combination, the QLattice was not constrained to any particular mathematical model, which ensures that all genes are put on equal footing. For each resulting 2-gene model, we calculated the AUC and scored the synergy according to *AAUC = AUC*_*Combinatim*_-*max(AUC*_*Gene*_*, AUC*_*Partngr*_)_***!***_ where *AUC*_*Combiriatian*_ is the AUC of the 2-gene model, *AUC*_*Gene*_ is the AUC of the 1-gene model for the current gene being held fixed, and *AUC*_*Partmr*_ is the AUC of the 1-gene model for the current partner gene.

It is important to note the significance of subtracting *max(AUC*_*Gene*_*, AUC*_Partner_) instead of simply *AUC*_*Csne*_ here. To exemplify this, in the case of the *BIRC5*-NEIL3 model from Fig. 3E. we find *AUC*_*BIRC5*_ = 0.50 and *AUC*_*NEIL3*_ = 0.51, whereas *AUC*_*BIRC5-NEILS*_ = 0.73, resulting in *ΔAUC* = 0,22. For comparison, with *LAMA1* (which is the most statistically significant gene based on p-value), we obtain *AUC*_*LAMA*1_ = 0.76 and *AUC*_*BIRC*5-*LAMA*1_ = 0.82. This would give the wrong impression that LAMA1 has great synergy with *BIRC5*, where in reality it is simply a consequence of the fact that LAMA1 is highly predictive on its own. Our *ΔAUC* measure is thus a true metric for finding the predictive increase *relative* to how strong the two genes are on their own.

Subsequent to this calculation for all 2-gene models, we rank, for each gene, the top 5 partner genes according to *AAUC*. and create a list of 5 × 102 = 610 synergistic partners. We note that although this list will contain many duplicates, the occurrence of gene X in the five best partners of gene Y does not guarantee the occurrence of gene Y in the five best partners of gene X. We then use StringDB^21^ to create a graph where each node corresponds to a gene, which will then have edges according to the amount of times it occurs in the list of synergy partners. Accordingly, the lowest possible node degree is 5, corresponding to the gene’s own five best partners, and every additional edge signifies the occurrence of the gene in the list of best partners for another gene.

We used a high confidence (0.700) and all interaction sources besides textmining in StringDB, which allowed us to identify three primary biological biological pathways. To visualize the correspondence of this synergy network to these established biological relations, we finally color each gene node according to which of the pathways they belong to.

### Regulatory Network Analysis

We used the expression data from all key genes across each comparison to perform an upstream regulator analysis. We identified the biological or chemical drugs in the QIAGEN IPA library of regulators, defined as having a *P* < 0.05 (Fisher’s Exact Test) and an absolute z-score > 1. Drugs from the data set that met these criteria and were associated with similar gene expression changes in the QIAGEN Knowledge Base were visualized by two-way hierarchical clustering of the unstandardized z-scores with Ward linkages in both dimensions.

## Figures and Tables

**Figure 6:**
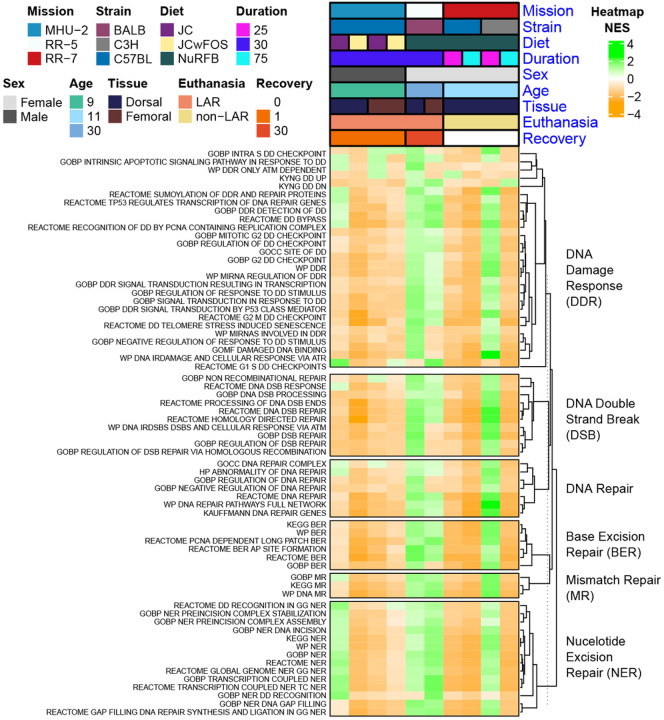
DNA damage and repair pathways being regulated in rodents flown to space. Heatmap of pathways relating to DNA damage response and repair mechanisms, highly significant (FDR ≤ 0.1) in at least 1 data subset.

## Data Availability

Many of the datasets used are publicly available via the NASA OSDR’s Biological Data Management Environment (https://osdr.nasa.gov/bio/repo), including murine skin RNA-Seq datasets (OSD-238, OSD-239, OSD-240, OSD-241, OSD-254), microarray data from the JAXA hair study (OSD-174), and the JAXA CFE data (OSD-530).
